# A Combinatorial Approach to Synthetic Data Generation for Machine Learning

**DOI:** 10.1007/s42979-025-04540-x

**Published:** 2026-01-07

**Authors:** Krishna Khadka, Jaganmohan Chandrasekaran, Yu Lei, Raghu Kacker, D. Richard Kuhn

**Affiliations:** 1 Department of Computer Science and Engineering, The University of Texas at Arlington, Arlington, TX 76019 USA; 2 National Security Institute, Virginia Tech, Arlington, VA 22203 USA; 3 Information Technology Laboratory, National Institute of Standards and Technology, Gaithersburg, MD 20899 USA

**Keywords:** Synthetic data generation, Combinatorial testing, Variational autoencoder, Differential privacy

## Abstract

Datasets used in machine learning often contain sensitive information, including personally identifiable health and financial details. A common challenge faced by organizations and researchers is the risk of privacy breaches when using real-world data. Synthetic data can be used as an alternative to the real-world data. In existing synthetic data generation techniques, an encoder processes the real-world data to map it into a lower-dimensional latent space. Random sampling is then performed in this latent space. Subsequently, a decoder network is utilized to generate synthetic data from these sampled points in the latent space. Such approaches typically require generating a large number of synthetic samples to approximate the performance of real-world data, subsequently slowing down downstream machine learning tasks. Addressing this, we introduce a combinatorial approach to sampling the latent space, motivated by our empirical findings within this study that most model predictions are largely influenced by interactions between a few features. In some cases, just using a small number of features produces accuracy better than using entire features. Through this approach, we generate samples that utilize t-way interactions among the t latent dimensions out of n. Our experimental results indicate that our approach requires fewer samples than traditional random sampling to achieve comparable model performance for real-world data sets. We also show that when integrated with a differentially private mechanism, our approach incurs a smaller decline in model performance than existing random sampling approach.

## Introduction

While real-world data is crucial for training machine learning models, privacy and legal restrictions prevent sharing datasets containing sensitive information. The use of real-world data presents two primary concerns: sensitive data can be exposed within the datasets themselves [1], and training models on this data can result in data leakages in the output [2]. Synthetic data generation, which creates artificial data resembling real-world data, offers a safer alternative. For example, synthetic data can be distributed instead of real data and used during the model training and evaluation phase. Doing so can significantly reduce the risk of exposing sensitive information.

Existing work on synthetic data generation techniques, such as Variational Autoencoders (VAEs), have been utilized in works by [3–5], etc. Similarly, Generative Adversarial Networks (GANs) have been employed in studies by [6–8], etc. These approaches utilize random sampling of the latent space to generate synthetic samples and have gained traction for their ability to produce synthetic dataset that closely mirror real-world dataset. It has been observed in this domain that random sampling strategy can result in uneven sampling in the latent space [9]. Moreover, achieving a machine learning performance close to real-world data requires a large number of synthetic samples [10]. In this paper, we address this problem by proposing a combinatorial sampling technique, extending methods used in combinatorial testing [11], to sample from latent space. As with software testing, our results suggest that individual model predictions are mainly affected by interactions between a few key features. We point out that the key features may be different for different model predictions, but the number of such features is small for most model predictions.

The main idea behind our approach is to systematically explore the latent space by covering all possible t-way interactions between the different latent dimensions, where t is a number that is typically much smaller than the total number of latent dimensions. Our approach is inspired by a widely used software testing strategy called t-way combinatorial testing or t-way testing [12]. Given a software system that takes k parameters as input, t-way testing creates a test set that covers all the t-way interactions, instead of all the k-way interactions, where t is typically much smaller than k. The key insight of t-way testing is that while the behavior of a system overall could be affected by many factors, individual faults are caused by interactions between only a few factors [12]. Empirical studies have shown that t-way testing can be very effective in fault detection while significantly reducing the number of test cases [12]. Our hypothesis is that, like software faults, many model decisions depend on interactions between just a few features. By capturing all the t-way interactions in the latent space, the synthetic data generated by our approach could capture most important patterns in the original training dataset. We provide empricial evidence that supports our hypothesis in Sect. 4.6.

Our approach consists of three major steps. First, we preprocess the training datasets and use an autoencoder network to learn the data’s latent space. Next, we utilize a discretization technique, which applies entropy-based discretization using the labels, to discretize the latent space. This ensures that we capture meaningful data partitions and also reduce the number of combinations in the latent space. Finally, these latent samples are passed through the decoder network to generate synthetic data, one data instance per latent sample.

We evaluated our approach’s effectiveness on various real-world datasets and models. We implemented our approach on real-world datasets, using a conditional Variational Autoencoder (cVAE) [13]. We also integrated our approach with the Differentially Private Stochastic Gradient Descent (DPSGD) algorithm to achieve differential privacy. Our approach was tested on the Travel Customer [14], Heloc [15], Adult Income [16], and Credit Default [17] datasets, and four distinct models: Linear Regression (LR), Decision Tree (DT), Random Forest (RF), and Support Vector Machines (SVM). Our approach maintained an accuracy within 5% of the real-world training dataset for 11 out of the 16 classifiers. Additionally, it achieved better statistical and privacy outcomes. When differentially privatized, our appraoch outperformed its random counterpart in 13 of the 16 classifiers. Our approach required significantly fewer samples than its random sampling counterpart.

In summary, we make the following contributions:

1. We conduct empirical studies that demonstrate the effectiveness of our approach. The synthetic dataset generated using our approach can achieve performance that is comparable to those derived from the real-world dataset. In addition, we show our approach achieves better statistical similarity than existing approaches that are based on random sampling.
2. We study the impact of t-way interactions of key features on model prediction. The results indicate that interactions among just a few key features can effectively determine model predictions without requiring the need for complete feature interaction.
3. We also show that our approach can be integrated with differentially private mechanisms, allowing for an additional layer of privacy protection without a significant drop in model performance compared to random sampling counterpart.

This paper is an extension to our earlier work [18], where we proposed the use of t-way combinatorial testing for sampling the latent space in synthetic data generation. In this paper, we extend our work with additional empirical evaluations, analysis of the impact of t-way sampling, and integration of differential privacy to generate differential private version of the dataset. The code used for our research is available on GitHub.^1^

The paper’s layout is: Sect. 2 covers background; Sect. 3 details our approach; Sect. 4 discusses experiments and results; Sect. 5 reviews related work; and Sect. 6 concludes and suggests future research avenues.

## Background

### T-way Combinatorial Testing

T-way combinatorial testing is commonly a software testing approach for generating tests that cover all the t-way interactions between parameters [11]. A t-way interaction is a combination of values involving t parameters, one value for each parameter. Table 1 provides an example of a 2-way test set for a system consisting of three parameters A, B, and C, each of which has two values, 0 and 1. This test set covers all the 2-way interactions as it covers all possible combinations between AB, AC, and BC. This test set does not cover all the 3-way interactions, which would require 8 test cases.

**Table 1:** An example 2-way test set

A	B	C
0	0	0
0	1	1
1	0	1
1	1	0

T-way testing has been shown to be highly effective for detecting software faults while significantly reducing the number of test cases. The key insight behind t-way testing is the following. While the behavior of a software system on the whole may be affected by many factors, many individual faults are caused by only a few factors since not every factor contributes to every fault. Thus, covering all the t-way interactions, instead of all possible interactions, could still detect a significant number of faults.

T-way testing can be considered a sampling strategy because it selects a subset of tests from the space of all possible tests. For instance, the 2-way test set in Table 1 selects four tests out of the possible eight tests. In the rest of the paper, we will refer to t-way testing as t-way sampling to be consistent with the terminology in the machine learning community. T-way sampling necessitates the identification of a set of parameters and a set of representative values for each parameter.

### Conditional Variational Autoencoder (cVAE)

cVAE is a generative model that expands upon the Variational Autoencoder (VAE) framework by incorporating conditioning information in the form of labels or other relevant features [13]. The cVAE architecture consists of two main components: an encoder network and a decoder network (a.k.a the generator). The encoder is responsible for compressing the input into a latent space representation, typically parameterized by mean and variance, assuming a normal distribution. The decoder then reconstructs the samples from this latent representation. The input data, X, is conditioned on the label, y, to guide the generative process. If the encoder and decoder are neural networks, then $\phi$ represents the weights of the encoder network, and $\theta$ represents the weights of the decoder network. The cVAE’s objective is to maximize the evidence lower bound (ELBO) by incorporating conditioning information, y, as illustrated in the following equation:

1 $$\begin{aligned} \mathcal {L}(\theta , \phi ; \textbf{x}, \textbf{y})&= \mathbb {E}_{q_\phi (\textbf{z}|\textbf{x}, \textbf{y})}[\log p_\theta (\textbf{x}|\textbf{z}, \textbf{y})] \nonumber \\&\quad - \text {KL}[q_\phi (\textbf{z}|\textbf{x}, \textbf{y}) || p(\textbf{z}|\textbf{y})] \end{aligned}$$

The first term, $\mathbb {E}{q\phi (\textbf{z}|\textbf{x}, \textbf{y})}[\log p_\theta (\textbf{x}|\textbf{z}, \textbf{y})]$, represents the reconstruction likelihood term, while the second term, $\text {KL}[q_\phi (\textbf{z}|\textbf{x}, \textbf{y}) || p(\textbf{z}|\textbf{y})]$, measures the Kullback–Leibler divergence between the learned distribution and the prior distribution.

### Differential Privacy

Differential Privacy (DP) is a mathematical framework ensuring that small changes in a dataset do not significantly affect the outcomes, protecting individual data privacy [19]. Mathematically, a model M is said to be ($\epsilon$, $\delta$) differentially private if for any datasets D and D’ differing by at most one item, and for any subset of outputs S, the following holds:

2 $$\begin{aligned} \Pr [M(D) \in S]&\le e^{\epsilon } \Pr [M(D') \in S] + \delta \end{aligned}$$

Pr is the probability of M producing an output in the subset S. A lower value of $\epsilon$ means higher privacy. If is $\epsilon$ 0 in an ($\epsilon$, $\delta$) model, then the mechanism provides complete privacy, but the resulting mechanism is not useful [20]. Hence, a tradeoff between complete privacy and utility is required [21].

#### Differentially Private Stochastic Gradient Descent (DPSGD)

Stochastic Gradient Descent (SGD) is an optimization technique in machine learning that iteratively calculates model parameters [22]. In deep neural network, SGD may unintentionally reveal private data details [23]. DPSGD, introduced by Abadi, combines DP’s privacy with SGD’s optimization by adding noise to gradient updates, ensuring individual data doesn’t overly sway the model’s learning. The core challenge of DPSGD lies in balancing data utility against privacy. The amount of noise added is a determinant of this balance, directly influencing the model’s final performance relative to the intended privacy level.

**Algorithm 1:**
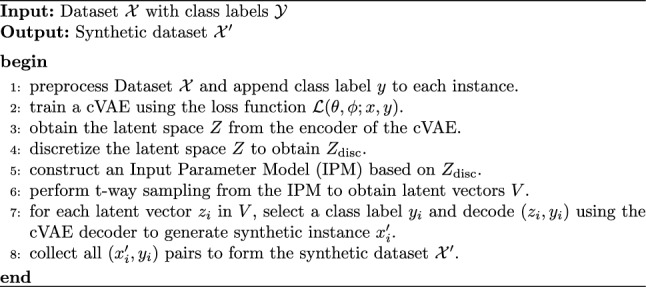
ComGen: Synthetic Data Generation

**Fig. 1:**
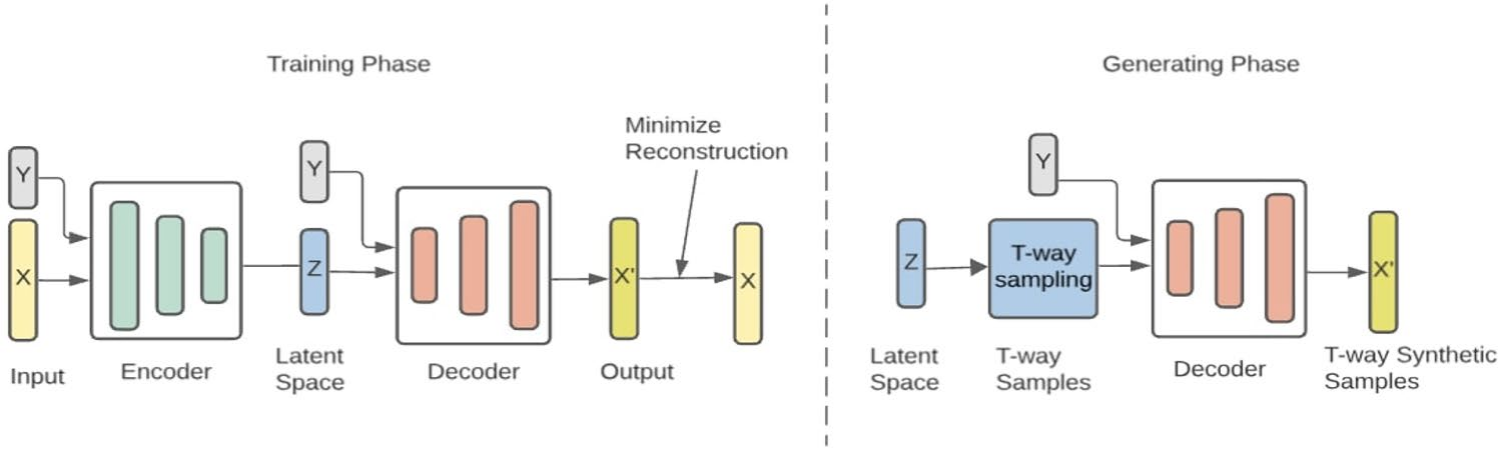
ComGen approach

## Approach

Our approach, **ComGen**, takes a real-world dataset *X* as input and produces a synthetic dataset *X*’ as output, which serves as a substitute to real-world training data. The generation of synthetic data involves obtaining the latent space of the original data using the encoder of the cVAE, discretizing the latent space, and generating a set of t-way latent vectors via CT. These vectors, together with their corresponding class labels, serve as input for the decoder of the cVAE to generate synthetic data. Figure 1 presents our approach.

**Note**: Our current implementation focuses primarily on tabular data, which includes numerical and categorical attributes. However, the approach can be adapted for other data types such as text, audio, and images with appropriate preprocessing steps. For example, text data might need tokenization and embedding, audio data could require spectral feature extraction, and image data might involve normalization and resizing. Extending our approach to these data types is a potential direction for future work.

### Step 1: Generation of Latent Space

In this step, the objective is to represent the data with fewer feature values that capture the essential properties of the original data. This is achieved using a latent, lower-dimensional representation of the data. The latent representation reduces data complexity, removes noise, and increases efficiency for data generation. To obtain this latent representation, we first train a cVAE on the dataset. Before training, the original data requires preprocessing, which may include one-hot encoding categorical attributes and normalizing numerical attributes. During training, the class label is added to both the encoder and the decoder by concatenating its one-hot representation with the input or latent vector. This allows the model to learn class-specific characteristics.

One challenge is to determine the number of latent dimensions. Latent dimensions is the number of columns in the latent space. An excess number of dimensions might introduce complexity and noise, while too few dimensions could lead to loss of information. A balance is needed to ensure proper representation. We address this challenge based on the dataset. For tabular data, given its structured nature with each dimension containing unique information, we begin latent dimension selection with half of the original dimensions. Then we explore the model loss within a particular range (for instance, [*h* + 4, *h* - 4]), where *h* represents half of the original dimension, to identify the latent dimensions yielding the smallest reconstruction error. This heuristic was experimented with and validated in our previous work conducted before the journal extension [18].

### Step 2: Generation of t-way Latent Vector

In this step, we generate t-way latent vectors using t-way sampling. First, we design the Input Parameter Model (IPM) based on the latent space derived from the previous step. For instance, if our latent dimension is seven, it denotes a reduction in the number of columns/features to this number after processing through the encoder network. The input dimension for the encoder network depends on input dataset.

Each column or feature in the latent space corresponds to a parameter within the IPM. As the latent space is continuous, we discretize it using an entropy-based discretization. This method helps in identifying representative values for every parameter in the IPM. Essentially, for each latent feature, we produce a set of discretized values. The identification of these discretized values involves determining bins or splits that offer maximum information gain. A deeper tree results in more values, allowing us to generate more samples. However, this comes with the trade-off of increased computational demands. For instance, consider Lime’s entropy-based discretization method: by default, it uses a tree depth of 3, which gives us up to 8 distinct values for each feature in the latent space [24].

After creation of the IPM, we apply t-way sampling to get t-way latent vectors. The value of ‘t’ determines the number of features engaged in the interaction. A higher ‘t’ value means more test instances. For instance, with 3-way sampling and seven latent features, our approach applies the interaction of three features to produce various combinations. These resulting combinations are t-way latent vectors. A significant challenge is to determine the optimal value of ‘t’. Based on our findings from RQ 1 in Sect. 4.6, which observed that model predictions remain stable with up to five feature interactions, we propose initially testing ‘t’ values from 2 to 5. This range is selected to ensure we capture significant interactions while maintaining computational efficiency. If increasing ‘t’ beyond 5 shows no significant gain in model performance, we will identify t = 5 as the optimal stopping point.

### Step 3: Generation of t-way Synthetic Samples

To generate t-way synthetic samples, we use the decoder part of the trained cVAE. It takes the t-way latent vectors together with the desired class labels and generates the synthetic data. Here, the class label is concatenated with the latent vector before decoding. The number of features in the newly generated sample matches that of the original input sample. Algorithm 1 represents our approach.

### Optional: Generation of Differentially Private t-way Synthetic Samples

Optionally, to generate differentially private synthetic samples, we propose **DP-ComGen**, which integrates a differential privacy mechanism with cVAE architectures leveraging DP-SGD.

During the training phase, DP-SGD introduces calculated noise into the model’s gradients, ensuring the differential privacy. The magnitude of this noise, and thus the level of privacy, is governed by the parameter $\epsilon$. A smaller $\epsilon$ indicates a stronger privacy guarantee, while a larger $\epsilon$ suggests weaker privacy. The relationship between $\epsilon$ and the noise introduced is crucial: a smaller $\epsilon$ will introduce more noise, potentially compromising model performance, while a larger $\epsilon$ will introduce less noise, potentially compromising privacy. Some binary classification works performed by [25] and [26] achieved high utility and privacy with $\epsilon$ close to 1. Based on these findings, we also choose an $\epsilon$ value of 1 for our purpose.

Post-training, we generate t-way latent vectors, representing t-way combinations of features within the latent space. These vectors along with class labels are then decoded using the cVAE’s decoder network, leading to the generation of differentially private synthetic samples.

### Example

We demonstrate our approach using the Adult Income dataset. This dataset is a binary classification set with 6 numeric and 7 categorical features. We split the dataset into an 80/20 ratio for training and testing. This resulted in 39,073 training instances and 9769 testing instances.

We used a Reversible Data Transforms (RDT) to preprocess the training data [27]. This library converts categorical features into numeric equivalents. We then normalized these features to ensure a consistent data scale. After normalization, we added the class label to the dataset, increasing the number of columns to 14.

The conditional Variational Autoencoder (cVAE) features an encoder-decoder structure with two hidden layers in each network. The class label is provided as a conditional input to both the encoder and the decoder. During training, the encoder uses both the data and the class label to learn a latent representation, while the decoder is conditioned on the same class label to generate samples that correspond to the specified class. This allows the cVAE to learn a shared distribution for variations in different classes. During data generation, we pair a latent vector sampled from the shared distribution with a desired label, and then feed the pair to the decoder. This ensures that the generated instance aligns with the desired class.

We started with a latent dimension of 7, which is half of the input dimension. By testing latent dimensions from 3 to 11, we found that a dimension of 4 minimized the reconstruction error. This indicates optimal preservation of the original features. The class label is added as an additional feature.

The cVAE encoder outputs a latent representation with four features per instance. We applied an entropy-based discretization with a tree depth of 3. This produced discretized features with 7, 7, 4, and 6 across the latent features.

Utilizing these features, we constructed an Input Parameter Model (IPM), which was input into the t-way test generation tool such as ACTS. We configured ACTS to apply 3-way interactions among features, generating a total of 1176 three-way latent vectors. These latent vectors were appended with class labels and passed through the decoder. This process generated 1176 synthetic samples conditioned on the class label.

To generate synthetic data using a differentially private model, we added noise to the gradients during the training of the cVAE using the DP-SGD algorithm. The same generation process was then repeated using this differentially private model, ensuring that the resulting synthetic data were produced by a model that satisfies differential privacy guarantees.

## Experiments and Results

We want to investigate the following three research questions:

1. RQ1: How do t-way interactions of important features impact the model prediction?
  Using the t-way approach, we investigated the influence of ‘t’ features on our model predictions. First, we trained a decision tree model. We then used SHAP values from a logistic regression model to rank features by their importance. The top ‘k’ features were identified by sorting these values. We then retrained our model using only these top ‘k’ features to check if the model’s predictive accuracy is retained. For the features not in the top ‘k’, we nullified their effect using the mean feature value.
2. RQ2: How effective is our t-way sampling approach, ComGen, in generating synthetic samples in comparison with existing approaches based on random sampling?
  We trained models on real and synthetic datasets produced by our t-way sampling and compared them to those from random sampling approaches. Evaluation metrics included model performance, statistical similarity between real and synthetic data distributions, and privacy cost.
3. RQ3: How effective is our t-way sampling approach when integrated with a differentially private model, DP-ComGen, in comparison with existing approach based on random sampling (VAE)?
  Integrating our t-way sampling with a differentially private model, we compared its performance against traditional random sampling with privacy mechanisms. Metrics included model accuracy, data distribution fidelity, and privacy cost.

### Datasets

We utilized with four datasets: Travel Customer [14], Heloc [15], Adult Income [16], and Credit Default [17] datasets, which consist of 954, 10,459, 44,842 and 30,000 samples, respectively. All these datasets have class labels represented as 0 and 1. Each dataset was split into train and test sets with an 80:20 stratified split, ensuring equal class representation. Categorical features in tabular data were one-hot encoded and the entire dataset was normalized. More details about the datasets are presented in Table 2.

**Table 2:** Information on tabular datasets

Dataset	Samples	Training size	Testing size	Numeric attrs	Categorical attrs	Classes
Travel customer	954	763	191	2	4	2
Heloc	10,459	8367	2092	23	0	2
Adult income	48,842	39,073	9769	6	7	2
Credit default	30,000	24,000	6000	23	0	2

### Data Generation Baselines

We evaluated our approach against several state-of-the-art: Tabular Variational Autoencoder (TVAE) [28], and CopulaGAN [27]. We adopted these approaches directly from the SDV framework without any alterations.

### Evaluation Metrics

To evaluate the performance of ComGen in comparison with other state-of-the-art models, we use a set of metrics. These metrics test the machine learning performance, statistical similarity between the synthetic and real-world dataset, and determine the privacy cost of the generative model.

#### Evaluating Machine Learning Performance

We divided each dataset into a training set and a test set. The training set was used to generate synthetic datasets. The performance of these synthetic datasets was then assessed using the test set, which originated from the original dataset. We applied two performance metrics: accuracy and F1 score. Accuracy is calculated as the total number of correct predictions divided by the total number of predictions. The F1 score, on the other hand, is the harmonic mean of precision and recall. Higher scores in both metrics indicate a higher quality synthetic dataset. These metrics were calculated using functions provided by scikit-learn [29].

Our classifiers, including Logistic Regression (LR), Decision Tree (DT), Random Forest (RF), and Support Vector Classifier (SVC), were all implemented using scikit-learn [29].

#### Statistical Similarity

To measure the statistical similarity, we used Kullbak Leibler (KL) Divergence and Kolmogorov–Smirnov(KS) between real-world data and synthetic data [30]. KL Divergence calculates the difference in marginal probability mass functions (PMF) for each variable in both datasets, providing an asymmetric measure of distance. A KL divergence of zero indicates identical distributions. The KS test, on the other hand, measures the symmetric disparity between the empirical cumulative distributions of continuous variables. For enhanced clarity and comparability in this study, all metric scores, including those from KL divergence and KS test, are normalized to a [0, 1] range. Specifically, the KL divergence score is standardized as 1/(1+ KL Divergence), with values nearing 1 signifying greater similarity between the datasets. Both KLD and KS scores were calculated using SDV metrics [27].

#### Privacy Cost

For privacy cost evaluation, we use SDV metrics to assess the risk of inference attacks. We use the NumericalMLP metric for continuous values and the CategoricalKNN metric for categorical values [30]. These assume that an attacker already possess a few columns of real data; they will combine it with the synthetic data to make educated guesses. The outcomes of these metrics are normalized, providing scores that range between 0 and 1, with 0 indicating maximum vulnerability and 1 indicating complete safety against inference attacks.

### Implementation

We built cVAE model consisting of two-layer encoder and decoder networks. We used LIME for continuous latent distribution discretization and used ACTS 3.2 for generating t-way latent vectors [31]. Opacus ensured differential privacy for our PyTorch-based cVAE models. Experiments were conducted on a MacBook Pro with an 8-core Apple M1 chip and 8GB RAM.

### Hyperparameters

For determining the latent dimensions, we started with an approximation equivalent to half of the original data dimensions. We conducted experiments within a proximate range of [h + 4, h - 4], aiming to find latent dimensions that yielded the smallest reconstruction error. As a result, the best latent dimensions were determined to be 4 for Travel Customer, 7 for both Heloc and Credit Default, and 6 for Adult Income.

For $t$-way interactions, we explored values ranging from $t = 2$ to $t = 5$ across all datasets. Our experimental results, as shown in Table 3, indicate that most model predictions for the given datasets rely on interactions involving up to 5 features.

Regarding differential privacy within the cVAE framework, we maintained the $\epsilon$ parameter at a value of 1 to balance data privacy and utility. All generative models were trained over 300 epochs. To ensure the validity and consistency of our results, we repeated the entire procedure thirty times and reported the averages across all experiments.

### Results and Discussion

***RQ1:*** In RQ1, we perform empirical evaluations to learn how t-way interactions of important features impact the model prediction.

Table 3 shows that a limited set of key features influences the majority of model predictions. For the ‘Adult Income’ dataset, with its 14 features, the cumulative effect of the top 4 features accurately predicted 7948 out of the 9769 test instances, which translates to 81.4% of the original predictions. Similarly, in the ‘Customer Travel’ dataset, which has 6 features, the top 3 features were enough in accurately predicting 159 out of the 191 test instances, amounting to about 83.2%. The ‘Credit Default’ dataset, with its 23 features, saw the top 4 features accurately predicting 4822 out of the 6000 test instances, or 80.4%. Lastly, the ‘Heloc’ dataset, with 24 features, had 1643 out of 2092 test instances (or 78.5%) accurately predicted by considering the top 4 features.

The results show that the model’s decision-making process is driven by the interactions of few features. To determine optimal value of k, which represents the number of key features that most influence the model’s predictions, our experiments indicate that 4 or fewer features are sufficient to preserve the majority of the original predictions. Additionally, the accuracy achieved with few set of features is close to the accuracy of the complete feature set, typically within a 1 or 2 percentage point difference. In the case of the ‘Heloc’ dataset, the top 4 features surpass the accuracy of the full feature set which highlights the potential benefit of using a reduced number of influential features. To validate this, we conducted an experiment where the decision tree model was initially trained with the full set of features, achieving perfect training accuracy of 1.00 but a lower test accuracy of 0.64. This significant disparity between training and testing performance clearly illustrated overfitting.

**Table 3:** T-way interaction for retaining model prediction

Dataset	Total features	Test instances	k=2	k=3	k=4	k=5	k=6
Adult Income	14	9769	7901	39	8	2	–
Travel Customer	6	191	154	5	1	–	1
Credit Default	23	6000	4487	253	50	32	–
Heloc	24	2092	1348	169	126	29	–

*k* = *t* represents the number of instances correctly predicted when using only *t* features

**Table 4:** Model performance comparison accuracy and standard deviation

Dataset	Model	#	Real-world	TVAE	CopulaGAN	DP-VAE	ComGen			DP-Comgen		
							t	#	Accuracy	t	#	Accuracy
Travel customer	LR	763	$0.84 \pm .036$	$0.79 \pm .025$	$0.76 \pm .018$	$0.74 \pm .029$	4	950	$0.82 \pm .009$	2	52	$0.72 \pm .027$
	DT		$0.84 \pm .041$	$0.76 \pm .023$	$0.67 \pm .030$	$0.72 \pm .022$	2	58	$0.84 \pm .013$	3	270	$0.8 \pm .032$
	RF		$0.85 \pm .052$	$0.79 \pm .020$	$0.76 \pm .020$	$0.74 \pm .018$	4	950	$0.83 \pm .018$	2	52	$0.8 \pm .015$
	SVM		$0.87 \pm .025$	$0.76 \pm .018$	$0.76 \pm .021$	$0.76 \pm .016$	4	950	$0.82 \pm .008$	3	270	$0.81 \pm .010$
Heloc	LR	8367	$0.72 \pm .021$	$0.68 \pm .008$	$0.66 \pm .061$	$0.59 \pm .035$	2	60	$0.68 \pm .021$	3	555	$0.66 \pm .031$
	DT		$0.64 \pm .018$	$0.67 \pm .012$	$0.56 \pm .016$	$0.62 \pm .042$	2	60	$0.7 \pm .017$	4	4320	$0.67 \pm .042$
	RF		$0.73 \pm .008$	$0.71 \pm .008$	$0.68 \pm .009$	$0.57 \pm .012$	3	462	$0.7 \pm .009$	3	554	$0.69 \pm .039$
	SVM		$0.67 \pm .014$	$0.59 \pm .020$	$0.55 \pm .025$	$0.6 \pm .016$	4	3476	$0.64 \pm .013$	3	554	$0.64 \pm .023$
Adult income	LR	39,073	$0.83 \pm .027$	$0.79 \pm .018$	$0.77 \pm .016$	$0.75 \pm .018$	2	64	$0.8 \pm .032$	3	390	$0.78 \pm .030$
	DT		$0.79 \pm .020$	$0.77 \pm .009$	$0.69 \pm .008$	$0.76 \pm .008$	4	3370	$0.78 \pm .025$	3	390	$0.75 \pm .025$
	RF		$0.85 \pm .017$	$0.82 \pm .009$	$0.76 \pm .005$	$0.76 \pm .01$	4	3370	$0.8 \pm .018$	3	390	$0.75 \pm .041$
	SVM		$0.8 \pm .008$	$0.78 \pm .004$	$0.76 \pm .003$	$0.73 \pm .012$	4	3370	$0.79 \pm .019$	4	2190	$0.76 \pm .032$
Credit default	LR	24,000	$0.81 \pm .012$	$0.8 \pm .005$	$0.77 \pm .021$	$0.77 \pm .025$	3	348	$0.81 \pm .031$	5	13,794	$0.77 \pm .024$
	DT		$0.79 \pm .003$	$0.76 \pm .016$	$0.69 \pm .030$	$0.61 \pm .016$	2	64	$0.77 \pm .014$	3	454	$0.71 \pm .011$
	RF		$0.82 \pm .006$	$0.8 \pm .021$	$0.76 \pm .006$	$0.75 \pm .030$	3	348	$0.81 \pm .023$	3	454	$0.78 \pm .009$
	SVM		$0.78 \pm .013$	$0.77 \pm .011$	$0.76 \pm .004$	$0.77 \pm .021$	4	3382	$0.79 \pm .011$	3	454	$0.78 \pm .020$

# = Number of Training Instances

**Table 5:** Model performance comparison F1 score and standard deviation

Dataset	Model	#	Real-world	TVAE	CopulaGAN	DP-VAE	ComGen			DP-Comgen		
							t	#	F1 Score	t	#	F1 Score
Travel customer	LR	763	$0.77 \pm .031$	$0.73 \pm .033$	$0.66 \pm .024$	$0.58 \pm .036$	4	950	$0.75 \pm .038$	2	52	$0.68 \pm .042$
	DT		$0.75 \pm .047$	$0.75 \pm .029$	$0.67 \pm .033$	$0.65 \pm .024$	2	58	$0.74 \pm .033$	3	270	$0.68 \pm .036$
	RF		$0.77 \pm .045$	$0.76 \pm .030$	$0.69 \pm .024$	$0.60 \pm .019$	4	950	$0.77 \pm .039$	2	52	$0.67 \pm .035$
	SVM		$0.69 \pm .010$	$0.65 \pm .024$	$0.65 \pm .011$	$0.58 \pm .036$	4	950	$0.66 \pm .029$	3	270	$0.65 \pm .023$
Heloc	LR	8367	$0.73 \pm .012$	$0.66 \pm .045$	$0.64 \pm .034$	$0.60 \pm .028$	2	60	$0.69 \pm .032$	3	555	$0.66 \pm .037$
	DT		$0.69 \pm .008$	$0.67 \pm .015$	$0.55 \pm .034$	$0.59 \pm .021$	2	60	$0.68 \pm .023$	4	4320	$0.66 \pm .042$
	RF		$0.73 \pm .023$	$0.71 \pm .010$	$0.67 \pm .013$	$0.58 \pm .009$	3	462	$0.70 \pm .018$	3	554	$0.68 \pm .031$
	SVM		$0.61 \pm .014$	$0.55 \pm .064$	$0.46 \pm .032$	$0.50 \pm .037$	4	3476	$0.58 \pm .062$	3	554	$0.55 \pm .032$
Adult Income	LR	39,073	$0.75 \pm .037$	$0.77 \pm .021$	$0.69 \pm .018$	$0.60 \pm .017$	2	64	$0.77 \pm .022$	3	390	$0.64 \pm .018$
	DT		$0.79 \pm .044$	$0.78 \pm .024$	$0.68 \pm .019$	$0.65 \pm .011$	4	3370	$0.79 \pm .023$	3	390	$0.65 \pm .042$
	RF		$0.79 \pm .046$	$0.82 \pm .012$	$0.67 \pm .025$	$0.59 \pm .029$	4	3370	$0.78 \pm .034$	3	390	$0.69 \pm .021$
	SVM		$0.73 \pm .013$	$0.71 \pm .025$	$0.65 \pm .017$	$0.58 \pm .035$	4	3370	$0.72 \pm .048$	4	2190	$0.63 \pm .029$
Credit default	LR	24,000	$0.75 \pm .014$	$0.73 \pm .015$	$0.66 \pm .021$	$0.59 \pm .025$	3	348	$0.74 \pm .025$	5	13,794	$0.67 \pm .017$
	DT		$0.68 \pm .031$	$0.75 \pm .017$	$0.67 \pm .026$	$0.65 \pm .022$	2	64	$0.75 \pm .031$	3	454	$0.63 \pm .043$
	RF		$0.78 \pm .005$	$0.74 \pm .009$	$0.69 \pm .012$	$0.59 \pm .017$	3	348	$0.76 \pm .026$	3	454	$0.68 \pm .015$
	SVM		$0.74 \pm .010$	$0.68 \pm .013$	$0.65 \pm .015$	$0.58 \pm .021$	4	3382	$0.71 \pm .023$	3	454	$0.64 \pm .029$

# = Number of Training Instances

**Table 6:** Mann–Whitney U test *P*-value for model accuracies across datasets

Dataset	TVAE	CopulaGAN	DP-VAE	ComGen	DP-Comgen
Travel customer	0.027	0.026	0.028	0.053	0.029
Heloc	0.561	0.200	0.029	0.770	0.465
Adult income	0.146	0.029	0.029	0.234	0.029
Credit default	0.309	0.029	0.029	0.766	0.055

A *p*-value Greater than 0.05 Indicates Insufficient Evidence to Reject the Null Hypothesis

**Table 7:** Mann–Whitney U test *P*-value for model F1 scores across datasets

Dataset	TVAE	CopulaGAN	DP-VAE	ComGen	DP-Comgen
Travel customer	0.381	0.041	0.028	0.552	0.028
Heloc	0.309	0.110	0.029	0.381	0.189
Adult income	1.000	0.029	0.029	0.882	0.029
Credit default	0.557	0.057	0.029	1.000	0.042

***RQ2:*** In RQ2, we perform empirical evaluations, including machine learning performance, statistical similarity, and privacy cost to learn the effectiveness of ComGen.

*Machine Learning Performance (Tables* 4, 5, 6, *and* 7): From the range of *t* values tested (*t* = 2 to *t* = 5), we report results for the *t* value that resulted the highest accuracy and F1 scores. In Table 4, ComGen outperformed its counterparts, TVAE and CopulaGAN, in 13 out of 16 experiments by achieving higher accuracy. It showed similar performance to TVAE in one case, while TVAE performed better in two instances. In Table 5, ComGen outperformed TVAE and CopulaGAN in 11 out of 16 experiments by achieving higher F1 scores. It showed similar performance to TVAE in 2 cases, while TVAE achieved higher F1 scores in 3 instances.

We also evaluated whether the performance of models trained on synthetic data differs significantly from that trained on real data. We formulated the null hypothesis that there is no statistically significant difference in the accuracy and F1 scores between the two sets of models trained on synthetic data versus those trained on real data. To test this, we used the Mann–Whitney U test [32].

A *p*-value greater than 0.05 indicates that we fail to reject the null hypothesis, suggesting that the observed performance differences are not statistically significant. While this does not confirm that the synthetic and real data produce identical model behavior, it does suggest that there is insufficient evidence to conclude a meaningful difference in performance.

Tables 6 and 7 report the resulting *p*-values for model accuracy and F1 score, respectively. For accuracy, ComGen consistently yielded *p*-values above 0.05 across all datasets, indicating no statistically significant difference from models trained on real data. For F1 scores, ComGen similarly showed insignificant differences in most cases. These observations lead us to fail to reject null hypothesis.

ComGen’s efficiency is highlighted by its superior performance even with fewer training samples. Furthermore, ComGen’s predictions consistently remained within a 5% margin below real-world data, which can be attributed to t-way sampling of the latent space that generates samples capturing important feature interactions.

**Table 8:** Statistical similarity metric: KL-divergence

Dataset	TVAE	Copula GAN	DP-VAE	ComGen	DP-ComGen
Travel Customer	0.8	0.73	0.62	0.83	0.77
Heloc	0.85	0.68	0.7	0.88	0.79
Adult Income	0.89	0.75	0.84	0.91	0.83
Credit Default	0.93	0.7	0.8	0.87	0.81

**Table 9:** Statistical similarity metric: Kolmogorov–Simrnov test

Dataset	TVAE	Copula GAN	DP-VAE	ComGen	DP-ComGen
Travel customer	0.78	0.79	0.67	0.90	0.82
Heloc	0.89	0.77	0.76	0.91	0.84
Adult income	0.86	0.76	0.79	0.88	0.80
Credit default	0.90	0.83	0.83	0.87	0.79

**Table 10:** Privacy cost metric: numerical MLP

Dataset	TVAE	Copula GAN	DP-VAE	ComGen	DP-ComGen
Travel customer	0.06	0.1	0.07	0.12	0.18
Heloc	0.09	0.09	0.12	0.21	0.24
Adult income	0.3	0.28	0.3	0.28	0.32
Credit default	0.28	0.31	0.29	0.31	0.38

**Table 11:** Privacy cost metric: categorical KNN

Dataset	TVAE	Copula GAN	DP-VAE	ComGen	DP-ComGen
Travel Customer	0.27	0.3	0.36	0.3	0.33
Heloc	N/A	N/A	N/A	N/A	N/A
Adult Income	0.24	0.24	0.27	0.34	0.37
Credit Default	N/A	N/A	N/A	N/A	N/A

N/A indicates that the datasets do not contain any categorical columns

To evaluate Statistical Similarity and Privacy Cost in RQ 2 and 3, we identified the $t$ values that most frequently resulted in the best Accuracy and F1 scores for each dataset, as detailed in Table 4 and 5. For ComGen, optimal performance was achieved with $t = 4$ for Travel Customer, $t = 2$ for Heloc, $t = 4$ for Adult Income, and $t = 3$ for Credit Default. In the DP-ComGen configuration, optimal results were consistently obtained with $t = 3$ for Travel Customer, Heloc, Adult Income, and Credit Default.

*Statistical Similarity (Tables *8*and* 9): ComGen consistently scored above 0.83 in KL-Divergence across datasets, indicating a strong resemblance between synthetic and real-world data. This trend was also observed in the Kolmogorov-Smirnov Test, except for the ‘Credit Default’ dataset where TVAE had slightly better score. This suggests ComGen’s t-way sampling effectively preserves statistical properties.

*Privacy Cost (Tables *10*and* 11): ComGen secured higher privacy scores than its counterparts in both NumericalMLP and CategoricalKNN, suggesting it as a better alternative for privacy preservation than its counterparts. This is due to Comgen’s utilizing data discretization, which reduces information leakage.

***RQ3:*** In RQ3, we perform empirical evaluations, including machine learning performance, statistical similarity, and privacy cost to learn the effectiveness of DP-ComGen.

*Machine Learning Performance (Tables* 4, 5, 6, *and* 7): From the range of *t* values tested (*t* = 2 to *t* = 5), we report results for the *t* value that resulted the highest accuracy and F1 scores. For accuracy, the differentially private version of ComGen (DP-ComGen) outperformed DP-VAE in 13 out of 16 tests and achieved the same performance in the remaining two. The accuracy difference between our DP model and real data was smaller than that of the DP-VAE model, which uses a random sampling approach. For F1 scores, DP-ComGen performed better than DP-VAE in 14 tests, while DP-VAE performed better in one instance, and one instance had the same F1 score performance.

We used the Mann–Whitney U test to compare model performance on synthetic versus real data, testing the null hypothesis that no significant difference in performance between models trained on synthetic data and those trained on real data. As shown in Tables 6 and 7, DP-ComGen yielded *p*-values above 0.05 for the Credit Default dataset in accuracy and 0.189 for the Heloc dataset in F1 score. In these cases, we fail to reject the null hypothesis, indicating no statistically significant difference. In contrast, DP-VAE produced *p*-values below 0.05 across all cases, leading us to reject the null hypothesis and conclude that its performance differs significantly from models trained on real data.

*Statistical Similarity (Tables *8*and* 9): DP-ComGen consistently achieved higher KL-Divergence scores compared to its counterpart, DP-VAE. In the Travel Customer dataset, the difference was as high as 0.15. However, for the Kolmogorov-Smirnov test in Table 8, DP-VAE scored higher than DP-ComGen for the Credit Default dataset.

*Privacy Cost (Tables *10*and* 11): DP-ComGen outperformed its counterpart DP-VAE in all datasets across both the NumericalKNN and CategoricalKNN metrics, except in the Travel Customer dataset. In this case, DP-VAE had a better score of 0.36 compared to DP-ComGen’s score of 0.36 in the CategoricalKNN metric.

Compared to random sampling, t-way sampling of latent space in synthetic data generation offers better performance. This performance is attributed to the t-way interactions of latent features and the data discretization that ComGen applies. This ensures a statistically similar synthetic dataset with extra defense against potential data inference attacks.

## Related Work

Synthetic data generation has been an active research area in recent years. There are two major uses for synthetic data, including data augmentation as presented in [33] and [34] and data privacy protection as performed in [35] and [36]. Existing work on data privacy protection can be classified into two groups. In the first group, synthetic data instances are generated individually, e.g., by mixing-up with other instances in the original training dataset and/or a public dataset [37]. In the second group, the original training dataset is first mapped to a latent space from which samples are drawn to produce synthetic data.

We focus on the second type of work closely related to ours, and we find several significant contributions in the field of synthetic data generation. In [28], the TVAE (Tabular Variational Autoencoder) generates synthetic tabular data using a variation encoder. Likewise, CTGAN (Conditional GAN) [28] and CopulaGAN [27] offer Generative Adversarial Network (GAN)-based techniques for synthetic data generation, with CopulaGAN incorporating a CDF-based transformation applied by Gaussian Copulas for easier data learning. ED-GAN proposed in [38], combines elements of both VAEs and GANs for medical image generation. Deep learning has indeed developed tools like VAEs including [28, 39–42] and GANs including [43–45] for dataset-level generation. All these related works use random sampling.

## Conclusion and Future Work

In this paper, we presented an approach consisting of three major steps: learning the distribution of a real-world dataset using the appropriate generative model, generating a t-way set of latent vectors using combinatorial sampling, and generating a synthetic dataset by feeding the t-way latent vectors to the decoder network of the generative model. To enhance privacy, we also implemented a differentially private version of the our approach. Our experimental evaluation shows that the use of ComGen for machine learning tasks can achieve performance close to the use of real-world datasets, with higher statistical similarity and higher privacy protection, all while requiring fewer samples.

Discretization is crucial in identifying representative values for each parameter and significantly impacts the quality of generated t-way latent vectors. We aim to explore discretization techniques, like decile-based, frequency-based [29], and chi-merge [46], assessing their impacts on performance. Future work also includes broader evaluations across diverse datasets and models. In addition, we plan to further investigate ways to effectively integrate constraints in the latent space, aiming to better mimic real-world complexities.

## Data Availability

Data and code are available at: https://github.com/krishnakhadka200416/ComGen.

## Footnotes

1 https://github.com/krishnakhadka200416/ComGen
